# Effects of Laser Texture Oxidation and High-Temperature Annealing of TiV Alloy Thin Films on Mechanical and Antibacterial Properties and Cytotoxicity

**DOI:** 10.3390/ma11122495

**Published:** 2018-12-08

**Authors:** Yin-Yu Chang, Jia-Hao Zhang, Heng-Li Huang

**Affiliations:** 1 Department of Mechanical and Computer-Aided Engineering, National Formosa University, Yunlin 632, Taiwan; yinyu@nfu.edu.tw (Y.-Y.C.); nicesmall82118@yahoo.com.tw (J.-H.Z.); 2 School of Dentistry, China Medical University, Taichung 404, Taiwan; 3 Department of Bioinformatics and Medical Engineering, Asia University, Taichung 413, Taiwan

**Keywords:** Ti-V-O coatings, annealing, laser texturing, cell viability, tribological performance, antibacterial properties

## Abstract

Titanium dioxide and vanadium oxides have been applied extensively in industrial and medical fields. The objective of this study was to develop various composite structures of titanium and vanadium oxide (Ti-V-O) coatings on pure titanium through high-temperature annealing and laser texturing oxidation, separately; additionally, surface morphologies, tribological and hydrophilic properties, and antibacterial and biocompatibility abilities of these Ti-V-O coatings were evaluated. TiV alloy thin films were deposited on pure titanium and then annealed to form Ti-V-O coatings through thermal oxidation and laser texturing oxidation. Ball-on-disc wear tests and contact angle tests were conducted to evaluate the tribological properties and wettability of the coatings, respectively. The antibacterial activity of the coatings was estimated by SYTO9 nucleic acid staining with *Staphylococcus aureus* (Gram-positive bacteria). The cell cytotoxicity of the coatings was analyzed following the ISO 10995-5:2009 standard with human skin fibroblast cells. The Ti-V-O coatings, subjected to annealing at 700 °C, demonstrated higher hardness (Hv 1171) and a lower friction coefficient (0.6). The highest hardness (Hv 2711) and the lowest friction coefficient (0.52) were obtained for the Ti-V-O after laser surface texturing oxidation at 100 kHz. The oxide coating obtained from 100 kHz laser texturing oxidation exhibited the lotus effect because of its systematic textured microstructures, and displayed superhydrophobic surface properties. Compared with the unannealed TiV coating, both the samples with high-temperature annealing and laser surface texturing oxidation had excellent antibacterial properties to *Staphylococcus aureus*. However, the Ti-V-O thin films exhibited notable cell cytotoxicity. Although the cell viability on Ti-V-O coatings were not ideal, this study confirmed improvement in surface hardness, tribology, and antibacterial performance in Ti-V-O coatings, which may have potential for use in biomedical tools, devices, and equipment.

## 1. Introduction 

The surface properties of biomaterials are important for biomedical implants and medical instruments [1]. Pure titanium and Ti alloys are the most commonly used metals in biomedical materials [2], and naturally form a TiO_2_ oxidation layer on the material surface. Naturally formed TiO_2_ oxidation layers are thin despite their excellent chemical inertness. Overly thin TiO_2_ layers cannot protect the pure titanium and Ti alloy beneath the surface. Methods, such as surface treatment and coating, can cover pure titanium with a special oxide layer to enhance mechanical properties, including corrosion and abrasion resistance. Currently, surface treatment and coating are promising methods for improving the surface properties of pure titanium and Ti alloys.

Anodization, plasma electrolytic oxidation, and high-temperature annealing (HTA) are the common techniques to generate an oxide layer on the surface and protect the metal beneath. The generated TiO_2_ layer exhibits numerous advantages, including favorable mechanical properties, corrosion resistance, and biocompatibility. Despite anodization and plasma electrolytic oxidation being mature technologies which require short processing time, these methods entail using electrolytes that contain heavy metals and chemicals. If incorrectly disposed of, such electrolytes pose a critical threat to the environment. With the increasing prevalence of the green industry worldwide, environmentally friendly surface treatment methods are receiving increasing attention.

Laser surface texturing oxidation (LSTO) [3] is an environmentally friendly surface treatment method. Compared with conventional titanium alloy annealing, LSTO does not require large amounts of electrolytes or long heating and cooling times. LSTO is a rapid and non-pollutive process [3], and enables partial treatment on specified positions of materials. Furthermore, LSTO can create systematic microtextured patterns on the target surface, thereby increasing the surface capacity and abrasion resistance of machine components required in industrial applications [4,5,6]. Compared with conventional methods, LSTO is an outstanding surface treatment method [7]. 

Titanium aluminum vanadium alloy (Ti-6Al-4V) is commonly used in medical instruments. Although the created biomedical instruments may disperse aluminum and vanadium into the human body [8], causing possible health threats, small amounts of these metals can efficiently improve the mechanical properties (e.g., mechanical strength and toughness) of the Ti alloy in devices. Since Ti-6Al-4V exhibits excellent biocompatibility, it is commonly used in medical implants and instruments. Currently, 20–30% of medical instruments are manufactured using mainly Ti-6Al-4V [9]. 

In order to improve the surface properties of pure titanium, this study used cathodic arc deposition to coat Ti metal with a uniform and dense thin film composed of titanium and vanadium (TiV) alloy. The LSTO method and conventional HTA method were then used to conduct oxidation treatment on the TiV coating. Finally, this study analyzed the effect of these two thermal oxidation methods on the surface morphology, elemental composition, surface hardness, tribological performance, hydrophobicity properties, antibacterial properties, and cell cytotoxicity of the coatings. 

## 2. Materials and Methods

### 2.1. Sample Preparation

Cathodic arc deposition was used to produce a TiV alloy thin film using pure titanium (Ti) and TiV alloy (40 at. % of Ti and 60 at. % of V) cathode materials. During the deposition of TiV alloy coating, a Ti thin film was deposited as interlayer to ensure favorable adhesion strength between the TiV alloy coating and titanium substrate. Ar was introduced into the vacuum chamber to keep the environmental pressure of 3.33 Pa, and the cathode current was 70 A. The deposition temperature was set at 300 °C, and the bias voltage was −100 V. Subsequently, the transition layer (Ti/TiV) and the top TiV coating were deposited in sequence. One group of the TiV-coated samples was annealed through HTA using a high-temperature tube furnace; the samples were heated to three different temperatures (i.e., 500, 600, and 700 °C). After 1 h of HTA at a constant temperature, the samples were cooled to room temperature. Another group was treated by LSTO, and three different laser frequencies (100, 300, and 500 kHz) were used to perform LSTO on the TiV coating surface (laser scanning speed: 100 mm/s; laser power: 10 W; and laser pitch: 0.08 mm). In addition to forming an uneven texture, the high energy density LSTO generated a thin Ti-V-O oxide layer on the surface. 

### 2.2. Material and Mechanical Tests

The surface morphology of the Ti-V-O coating was examined using a laser scanning microscope (VK-X100, Keyence Co., Osaka, Japan), and a field emission scanning electron microscope (FESEM) (JSM-6700F, JEOL, Tokyo, Japan). The chemical composition of the deposited Ti-V-O films was confirmed through energy dispersive spectroscopy (EDS). In this study, a 52100 chromium steel ball was used to conduct experimental ball-on-disk abrasion test without lubrication by ball-on-disk tribometer (CH-2007, CSEM Instruments, Neuchatel, Switzerland). The tribological condition was: applied load = 1 N, sliding distance = 500 m, speed = 30 cm/s, and wear-track radius = 6 mm. The coefficient of friction (COF) of each Ti-V-O sample during the abrasion test was obtained. FESEM was used to observe the wear-track surface of the experimental samples, and control samples with the lowest COF. Furthermore, the EDS element mapping function of the SEM was employed to analyze the elemental composition of the wear-track surface.

The surface hardness and hydrophobicity of the samples were measured using a Vickers hardness testing machine (MMT-X, Matsuzawa Co., Ltd., Tokyo, Japan) and contact angle equipment (FTA-125, First Ten Angstroms, Portsmouth, VA, USA). For the hydrophobicity test, the obtained images were analyzed to calculate the contact angle of deionized water for each sample at room temperature. The data for surface hardness and contact angle are both expressed as mean and SD values for at least three independent measurements.

### 2.3. Antibacterial and Cytotoxicity Tests

The retention of bacteria on the coated samples was determined using a fluorescence staining method that employed a SYTO9 nucleic acid stain (Molecular Probes, Eugene, OR, USA). First, 500 μL of *Staphylococcus aureus* (2 × 10^7^ cfu/mL) was added to the sample surface. After incubation for 6 h at 37 °C under a relative humidity of 96% and the avoidance of light exposure, the sample surfaces were rinsed three times with phosphate-buffered saline; then, the retained bacteria were fixed with 4% paraformaldehyde (Sigma-Aldrich, St. Louis, MO, USA) and stained with 10 μM STYO9 for 30 min at room temperature. The bacteria that had adhered to the samples were quantified by measuring the fluorescence detected at 488 nm by using an enzyme-linked immunosorbent assay reader (Synergy HT, BioTek Instruments, Winooski, VT, USA). The results were quantified in units of relative fluorescence intensity. Statistical correlations in the results of antibacterial activity tests between each sample were determined using Student’s *t* test. Differences were considered significant at the 0.05 level.

Cytotoxicity tests were performed by following the ISO 10993-5 test for in vitro cytotoxicity using human skin fibroblast (HSF) cells purchased from Bioresource Collection and Research Center (product no. BCRC 60153, Bioresource Collection and Research Center, Hsinchu, Taiwan). The extraction medium of the test samples was prepared using Dulbecco’s modified Eagle’s medium (DMEM, Invitrogen, Carlsbad, CA, USA) supplemented with 10% serum and 1% antibiotics (Gibco BRL, Gaithersburg, MD, USA). The extract was based on the thickness of material extraction ratio of 1.25 cm^2^/mL ± 10%, and was extracted under the same conditions. Extracts for the test materials were based on 6-well plates, and every sample had, at most, 3.5 mL of medium, and was incubated at 37 °C under 5% CO_2_ in humidified air. The conditioned medium was collected after 72 h of culture and passed through a 0.2 μm filter. HSF cells (product No. BCRC 60153, Bioresource Collection and Research Center, Hsinchu, Taiwan) were seeded at a density of 5 × 10^4^ in 6-well polystyrene plates containing DMEM with 10% serum and 1% antibiotics, and incubated at 37 °C in the presence of 5% CO_2_. Cell cultures were incubated with samples or positive (DMEM) and negative (only medium) controls under the same conditions. After incubation overnight at 37 °C with 5% CO_2_ in humidified air, the culture medium was changed and then the sample was treated with extraction medium for 24 h. The test well contents were examined and recorded as microscopic images to determine cell morphology. Then, they conditional medium was changed again, and 1.2 mL/well 3-(4,5-dimethylthiazol-2-yl)-2, 5-diphenyltetrazolium bromide (MTT assay) was added. After reacting with MTT for 4 h, 1.2 mL of dimethyl sulfoxide (DMSO) was added to dissolve blue crystals in cells for 10 min, the supernatants were added at a concentration of 200 μL/well in a 96-well plate, and absorption at 570 nm was measured using a standard spectrophotometer to determine cell cytotoxicity.

### 2.4. Statistical Method

The statistical analyses of the results on wettability, surface hardness, antibacterial, and cytotoxicity tests between the coated samples and uncoated pure-Ti plates was determined by Student’s *t* test. Differences were considered significant at the *p* < 0.01 level.

## 3. Results and Discussion 

### 3.1. Surface Morphologies and Chemical Composition Analyses

**Figure 1:**
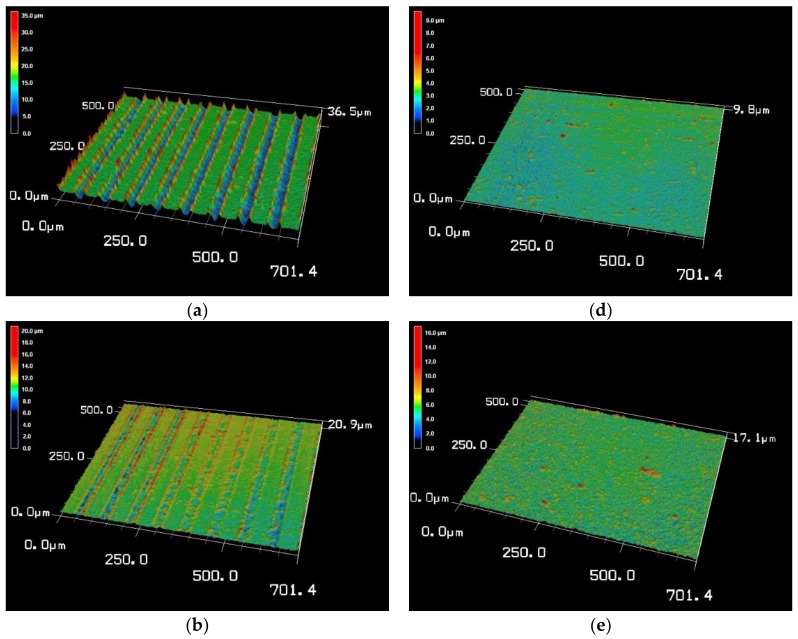

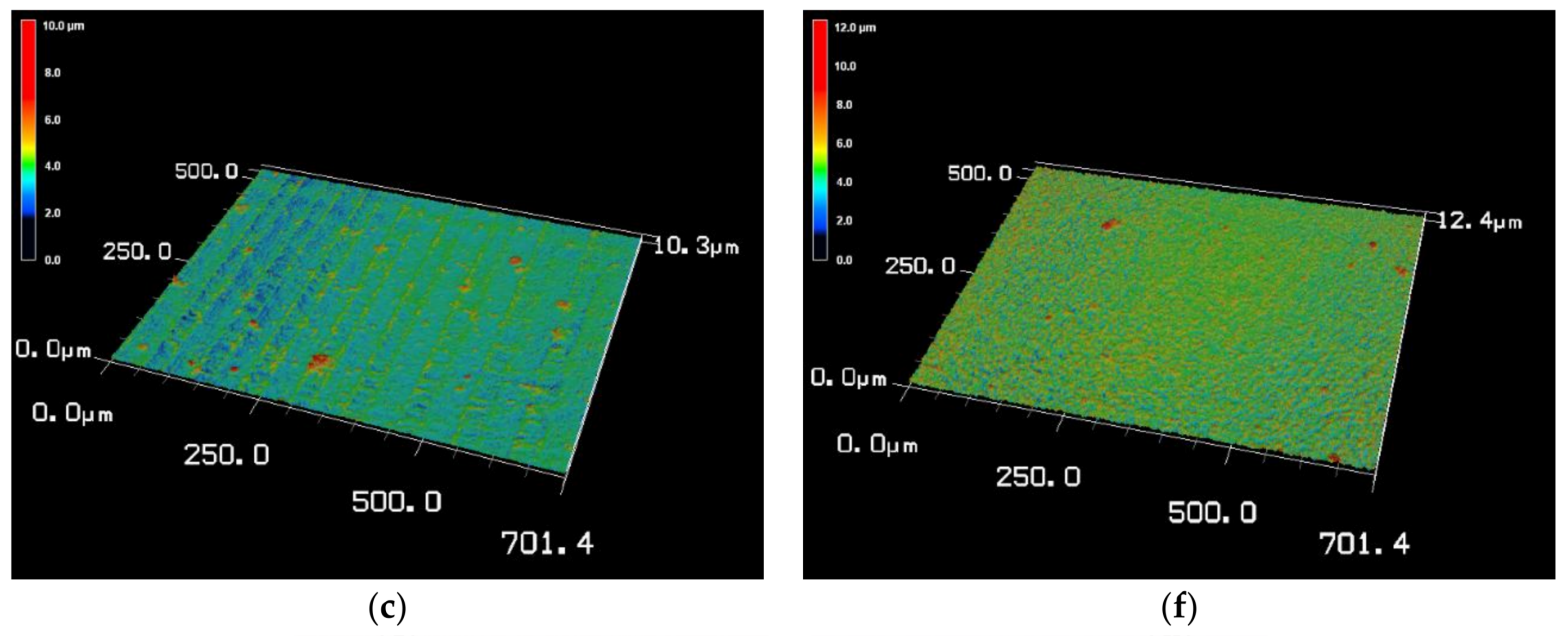
3D surface morphologies of the Ti-V-O coatings after laser surface texturing oxidation (LSTO) treatment at laser frequencies of (**a**) 100 kHz; (**b**) 300 kHz; and (**c**) 500 kHz; and Ti-V-O coatings after high-temperature annealing (HTA) treatment at (**d**) 500 °C; (**e**) 600 °C; and (**f**) 700 °C.

Figure 1 displays the 3D surface morphology of Ti-V-O coatings using a laser scanning microscope. Results revealed that the surface morphology and texture of the samples exhibited obvious changes when oxidized after HTA and LSTO. Low laser frequency possessed high laser energy density, creating greater distinction between laser-processed areas and untreated areas. When the laser frequency was 100 kHz, the coating exhibited noticeable surface morphological changes (Figure 1a), particularly, changes in texture depth and protrusions. A higher laser frequency is attributed to more flashes from the light source, which, in turn, shortens the time used to focus laser energy on the surface. Of the laser settings used to perform LSTO on the TiV coatings, the highest laser frequency was 500 kHz; hence, the coating produced using this frequency was exposed to the least amount of energy density and, therefore, exhibited the least notable morphological changes. For the samples treated by HTA, as shown in Figure 1d, the Ti-V-O coating did not exhibit noticeable morphological differences after undergoing annealing at 500 °C. However, after annealing at 600 °C, the coating surface exhibited changes in roughness; in addition to grain enlargement, the coating surface developed an oxide layer. When the annealing temperature was increased to 700 °C, the increasing amount of oxides created an obvious difference in the surface morphology of the coating.

**Figure 2:**
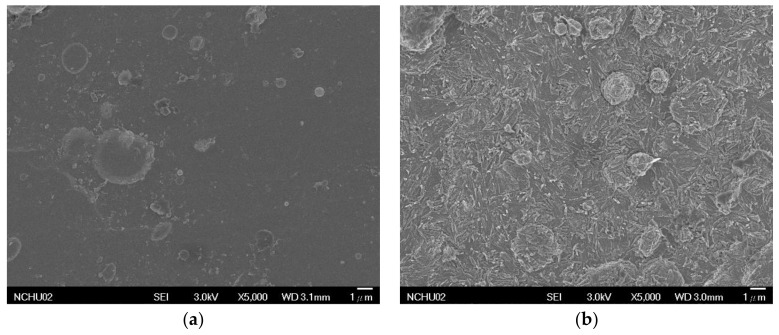

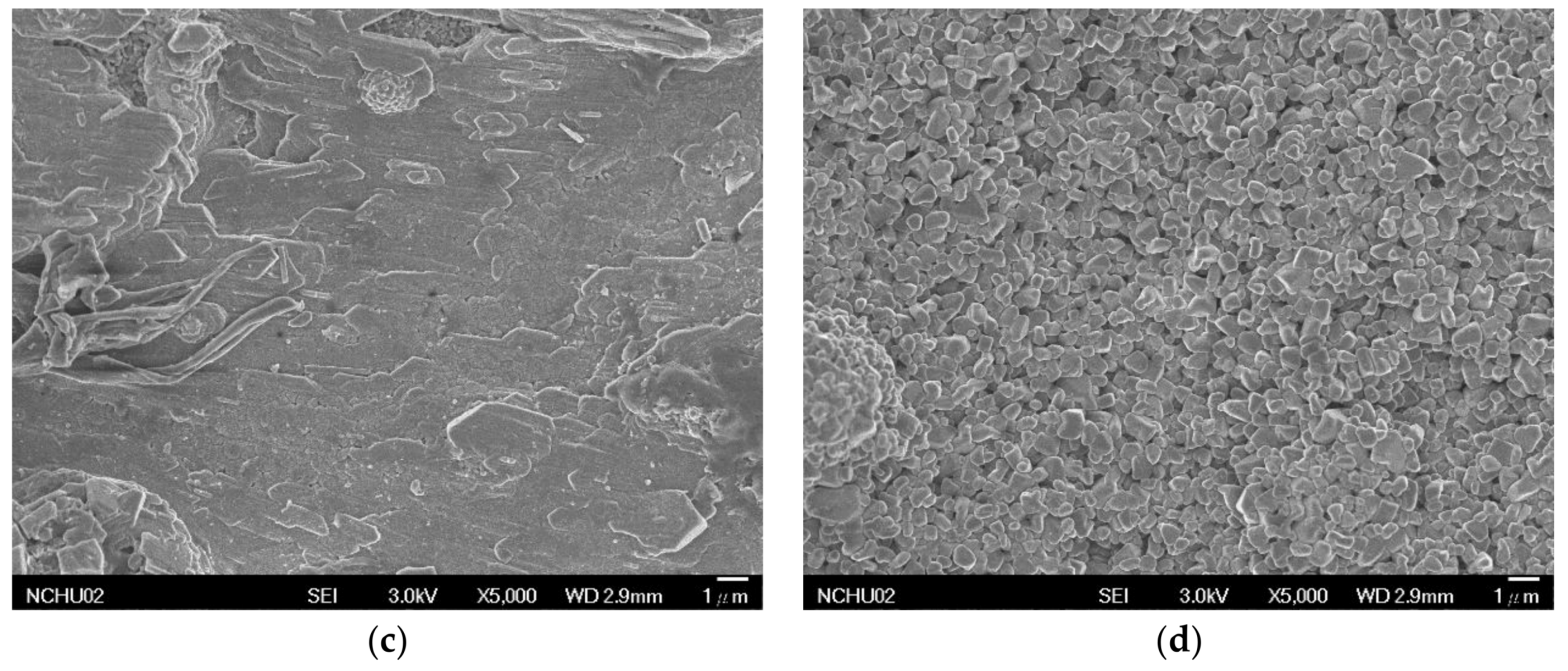
Surface morphology of the TiV coatings and the Ti-V-O coatings following HTA. (**a**) TiV; (**b**) Ti-V-O 500 °C; (**c**) Ti-V-O 600 °C; (**d**) Ti-V-O 700 °C.

Figure 2 shows the SEM images of the Ti-V-O coatings after HTA treatment. The untreated TiV coating exhibited some microparticles on the surface, due to the evaporation of TiV droplets on the samples, as shown in Figure 2a. When HTA was conducted at 500 °C, the Ti-V-O coating exhibited blob- and needle-shaped oxides (Figure 2b); under 600 °C, the oxide changed to stripe-shaped (Figure 2c). The EDS results (Table 1) showed that when the content of oxygen in the Ti-V-O coating increased to 57.21%, the vanadium content did not exhibit a considerable change, but the titanium content lowered to 10.41%. This indicated that after oxidation at 600 °C, the Ti-V-O coating mainly consisted of vanadium oxide (V_2_O_5_). After the oxidation temperature was increased to 700 °C, the Ti-V-O coating exhibited grain-shaped oxides (Figure 2d). This result was similar to that of Guo et al. [10] who reported that, at approximately 656 °C, TiO_2_ thin films produced grain-shaped oxides. Furthermore, the results of the present study revealed that the oxygen increased to 64.43%, the titanium content increased from 10.41% to 31.29%, whereas the vanadium content decreased to 4.28%. That indicated that when the Ti-V-O coating sample was heated to 700 °C, it mainly consisted of TiO_2_.

**Figure 3:**
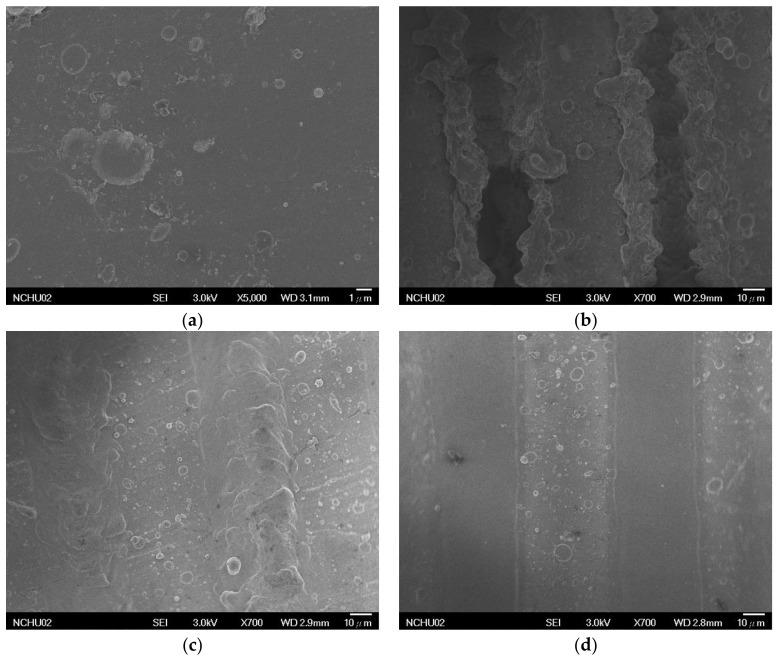
Surface morphology of the TV coatings and the Ti-V-O coatings following LSTO. (**a**) TiV; (**b**) Ti-V-O 100 kHz; (**c**) Ti-V-O 300 kHz; (**d**) Ti-V-O 500 kHz.

In the LSTO method, a low laser frequency led to high laser energy density; the texture depth and surface protrusions of the 100 kHz Ti-V-O sample were the most distinguishable (Figure 3). Additionally, increasing the laser energy density increased the working temperature, resulting in greater temperature differences between the surface and bottom of the sample. The annealing effect created under large temperature differences resulted in higher oxygen component ratios, thereby generating more surface oxides (Table 2).

### 3.2. Vickers Hardness Test

**Figure 4:**
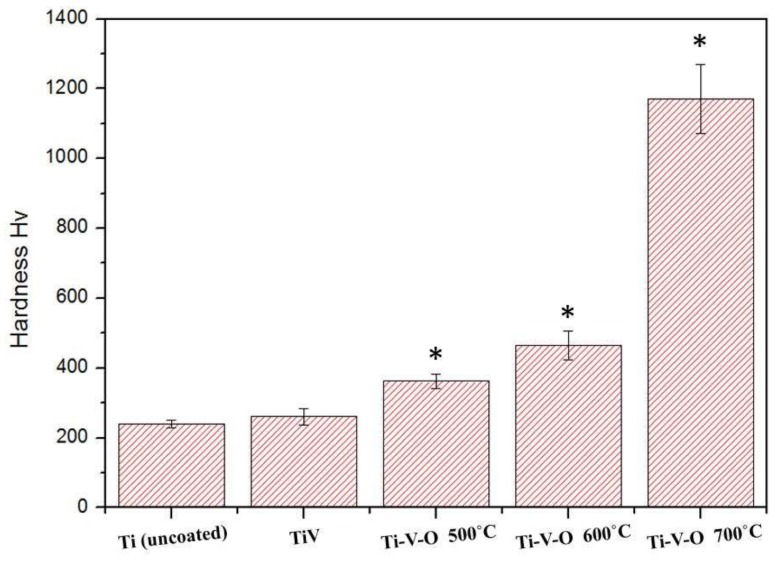
Vickers hardness test results of the uncoated Ti, TiV coating, and HTA Ti-V-O coating samples. * Significantly different from the control group—uncoated Ti plate (*p* < 0.01).

Results of the Vickers hardness test on Ti-V-O coatings after HTA are shown in Figure 4. As the annealing temperature was increased from 500 °C to 700 °C, the coating hardness increased from Hv 362 ± 50 to Hv 1171 ± 144. This result was similar to that of Zhang et al. [11] who demonstrated that in early annealing stages, the deposited coating released residual stress, and the hardness did not increase obviously. However, when the annealing temperature was further increased, the titanium alloy thin-film surface exhibited the effect of oxidation, substantially increasing thin film hardness.

**Figure 5:**
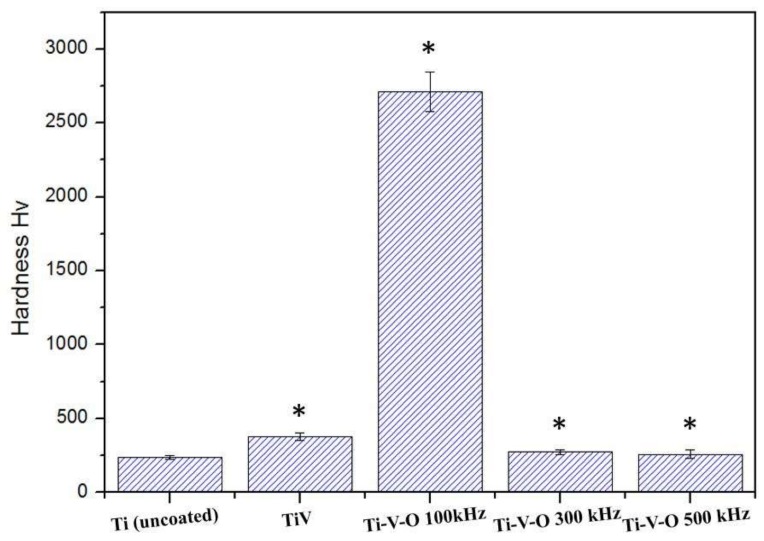
Vickers hardness test results of the uncoated Ti, TiV coating, and LSTO Ti-V-O coating samples. * Significantly different from the control group—uncoated Ti plate (*p* < 0.01).

Figure 5 displays the Vickers hardness test results of LSTO-treated coatings. The results revealed that TiV coatings had greater hardness than the Ti-V-O 300 kHz and Ti-V-O 500 kHz coatings. By contrast, the Ti-V-O 100 kHz coating (which was subjected to the highest level of laser energy density) exhibited a remarkable increase in hardness (2710.97 ± 136 Hv). Similar results were reported in the study of Montealegre et al. [12]. The results indicated that high laser frequency and scanning speed reduced the hardness of the sample; low laser frequency and scanning speed led to high sample surface temperature increase and quenching to room temperature, thereby increasing the hardness.

### 3.3. Ball-on-Disk Wear Test

**Figure 6:**
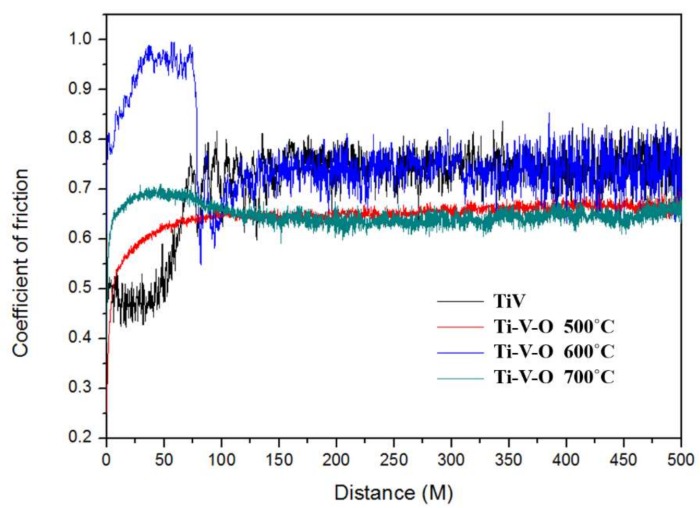
Coefficient of friction (COF) values of the TiV coating and HTA Ti-V-O coating.

**Figure 7:**
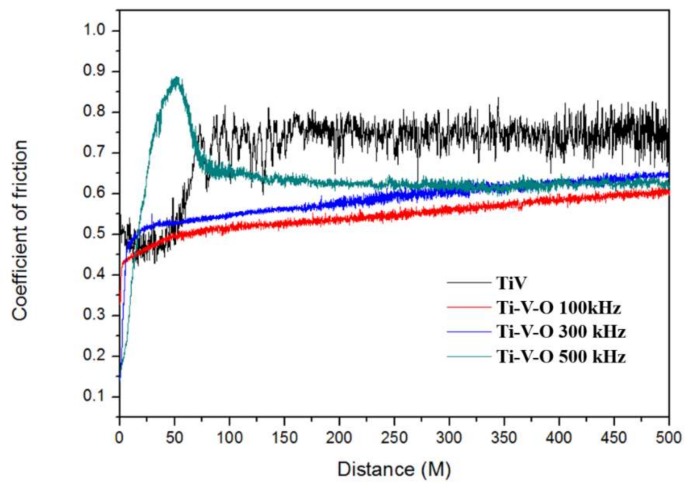
COF values of the TiV coating and LSTO Ti-V-O coating.

Results revealed that compared with untreated TiV coatings, HTA treated samples (Ti-V-O 500 °C, Ti-V-O 600 °C, and Ti-V-O 700 °C) all exhibited lower COF values, and the Ti-V-O sample annealed at 700 °C demonstrated the lowest COF of 0.6 (Figure 6). The results indicated that the HTA temperature influenced the COF of deposited coatings, and the COF value was reduced for the samples after HTA at higher temperature. The Ti-V-O coatings treated by the LSTO method (Figure 7) exhibited similar results. Compared with the untreated TiV coating, the Ti-V-O coatings treated by LSTO at frequencies of 100, 300, and 500 kHz exhibited lower COF values. Reducing the laser frequency transferred a greater amount of laser energy density to the deposited TiV coatings, generating more surface oxides and reducing the COF value; hence, the Ti-V-O samples with coatings deposited at a frequency of 100 kHz had the lowest COF value.

According to Ryk et al. [13], the surface COF values of LSTO coatings were lower than those of unannealed samples. The LSTO coating surface exhibited a textured structure with uniform patterns; hence, when a tribological load was applied, the surface served as a microreservoir for the oxides. The oxides stored within the surface structure keep a stable wear process and further improve the surface abrasion resistance.

### 3.4. Wettability Test

**Figure 8:**
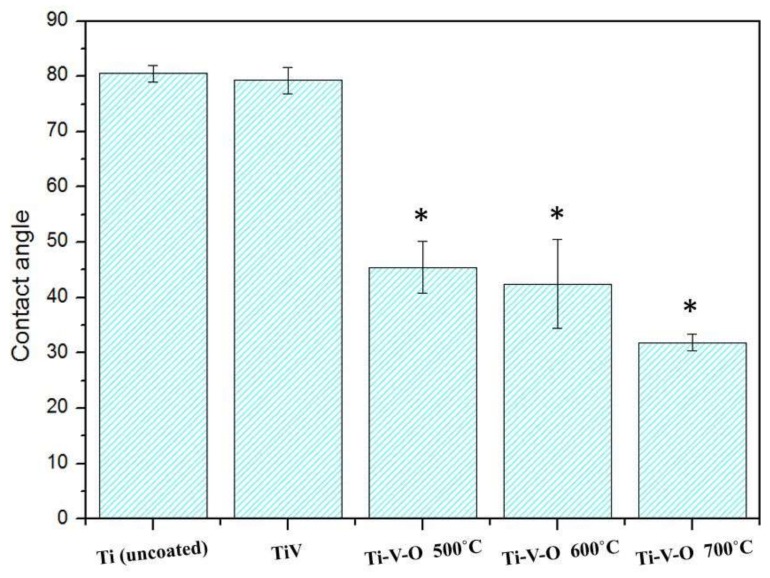
Water contact angle of the Ti (uncoated) sample, Ti-V-coated sample, and Ti-V-O-coated sample subject to HTA. * Significantly different from the control group—uncoated Ti plate (*p* < 0.01).

Figure 8 shows that HTA-treated Ti-V-O samples exhibited a lower water contact angle for the sample annealed at higher temperatures. The Ti-V-O coating annealed at 700 °C had the lowest water contract angle. When it was annealed at higher temperature, the surface of the deposited oxide layer had greater roughness, increasing its wettability. 

**Figure 9:**
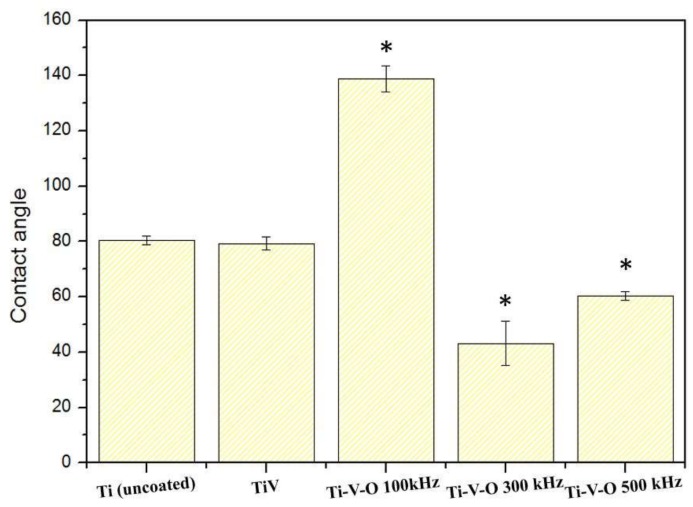
Water contact angle of the Ti (uncoated) sample, Ti-V-coated sample, and Ti-V-O sample subjected to LSTO. * Significantly different from the control group—uncoated Ti plate (*p* < 0.01).

Figure 9 shows that the 300 kHz LSTO coating and 500 kHz LSTO coating had greater surface roughness and wettability than did the Ti (uncoated) sample and the TiV deposited sample. However, the Ti-V-O coating treated under a laser frequency of 100 kHz had a high water contact angle of 140°, considerably reducing surface wettability and forming a superhydrophobic surface. When LSTO increased the surface roughness of the deposited coating, the uniform surface texture of the 100 kHz coating demonstrated the lotus leaf-like effect, achieving superhydrophobic properties. Yang et al. [14] conducted a water contact angle analysis on the striped, netted, and dotted surface of coatings subject to LSTO, and revealed that coatings composed of titanium, stainless steel (AISI 316L), aluminum alloy (A16061), and tungsten carbide also exhibited superhydrophobic surfaces. This result confirmed that surface superhydrophobicity is mainly affected by surface morphology instead of material properties.

### 3.5. Antibacterial Analysis

**Figure 10:**
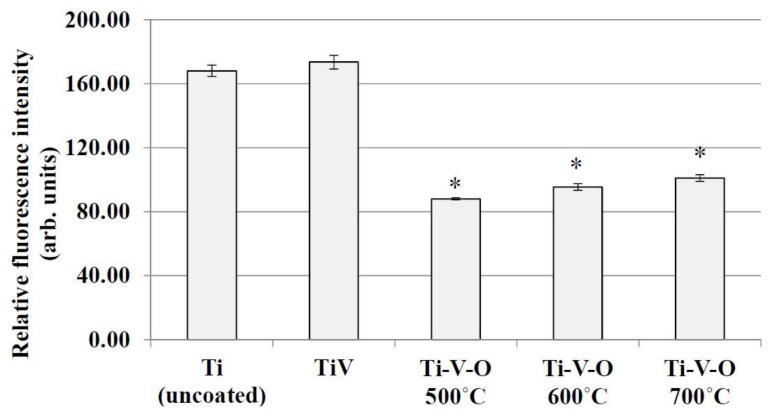
Antibacterial effects of the Ti (uncoated) sample, TiV coating, and HTA Ti-V-O coating. * Significantly different from the control group—uncoated Ti plate (*p* < 0.01).

**Figure 11:**
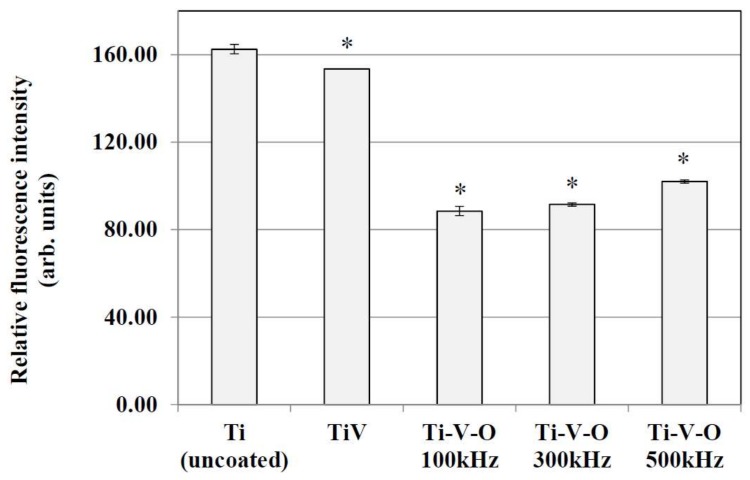
Antibacterial effects of the Ti (uncoated) sample, TiV coating, and LTSO Ti-V-O coating. * Significantly different from the control group—uncoated Ti plate (*p* < 0.01).

In this study, *Staphylococcus aureus* (Gram-positive bacteria) was used to analyze the antibacterial properties of samples coated with Ti-V-O using HTA and LSTO. The longitudinal axis of Figure 10 and Figure 11 is related to the amount of remaining bacteria, and a lower value represented greater antibacterial properties.

As shown in Figure 10, the generation of vanadium and titanium oxides caused the Ti-V-O 500 °C, Ti-V-O 600 °C, and Ti-V-O 700 °C HTA coatings to demonstrate excellent antibacterial properties. However, the difference in antibacterial values between those three samples were small. This indicated that although the HTA coatings exhibited different element compositions and roughness levels, as long as vanadium oxide (V_2_O_5_) was formed, the coatings exhibited observable antibacterial properties. V_2_O_5_ is an toxic properties [15,16], which resulted in Ti-V-O 500 °C, Ti-V-O 600 °C, and Ti-V-O 700 °C HTA coatings achieving similar antibacterial effects.

The LSTO Ti-V-O coating also demonstrated excellent antibacterial properties (Figure 11). Similar to HTA Ti-V-O coatings, the titanium oxide produced through LSTO had photoactive antibacterial effects [17,18], and the vanadium oxide produced also exhibited antibacterial properties [19]. By combining titanium oxide and vanadium oxide, the presented results coincided with the study of Wren et al., indicating that the Ti-V-O thin film exhibited greater antibacterial properties [20]. By contrast, the 100 kHz of LSTO Ti-V-O coating demonstrated higher antibacterial properties because of its superhydrophobic surface (i.e., exhibiting a water contact angle of 140°). In reference to Zhang et al. [11], when the coating surface came into contact with bacteria cells, the superhydrophobic surface lowered the binding power between the bacteria cell and sample surface. This reduced bacterial cells’ ability to attach to the sample surface, resulting in antibacterial effects. As a result of this, the 100 kHz LSTO Ti-V-O coating seems to exhibit superior antibacterial properties compared with the 300 kHz and 500 kHz LSTO Ti-V-O coatings.

### 3.6. Cytotoxicity Analysis

**Figure 12:**
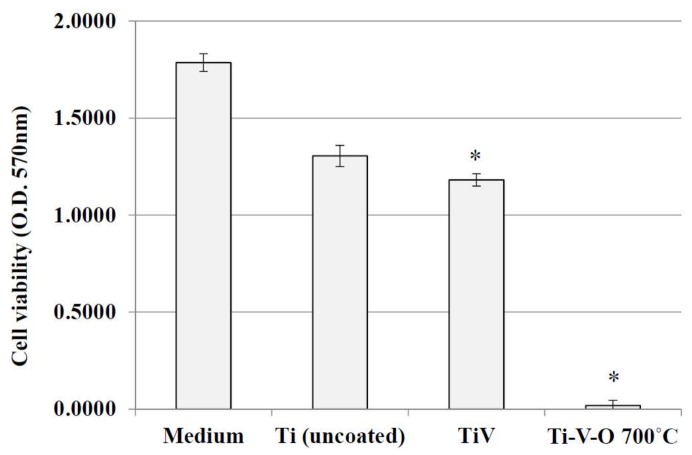
Cell viability of the extraction medium, Ti (uncoated) sample, TiV coating, and 700 °C HTA Ti-V-O coating. * Significantly different from the control group—uncoated Ti plate (*p* < 0.01).

**Figure 13:**
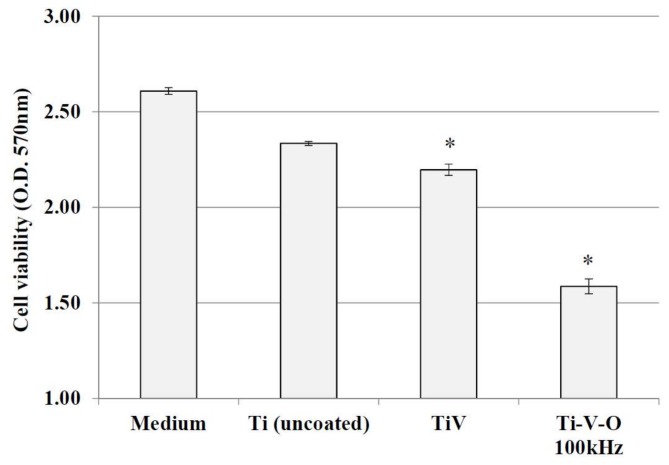
Cell viability of the extraction medium, Ti (uncoated) sample, TiV coating, and 100 kHz LSTO Ti-V-O coating. * Significantly different from the control group—uncoated Ti plate (*p* < 0.01).

Figure 12 and Figure 13 display the cytotoxicity effects of the 700 °C HTA-deposited coating sample and the 100 kHz LSTO-coated sample on HSF cells. Higher longitudinal axis values indicate greater cell proliferation. The dish with the extraction medium only seems to have the highest cell viability. Compared with the Ti (uncoated) sample, cell proliferation of Ti-V-deposited coated sample and the samples obtained from 700 °C HTA and 100 kHz LSTO were significantly lower. This verified the cytotoxicity of Ti-V-O coatings, particularly the 700 °C HTA Ti-V-O coating, which had minimal bacterial growth.

**Figure 14:**
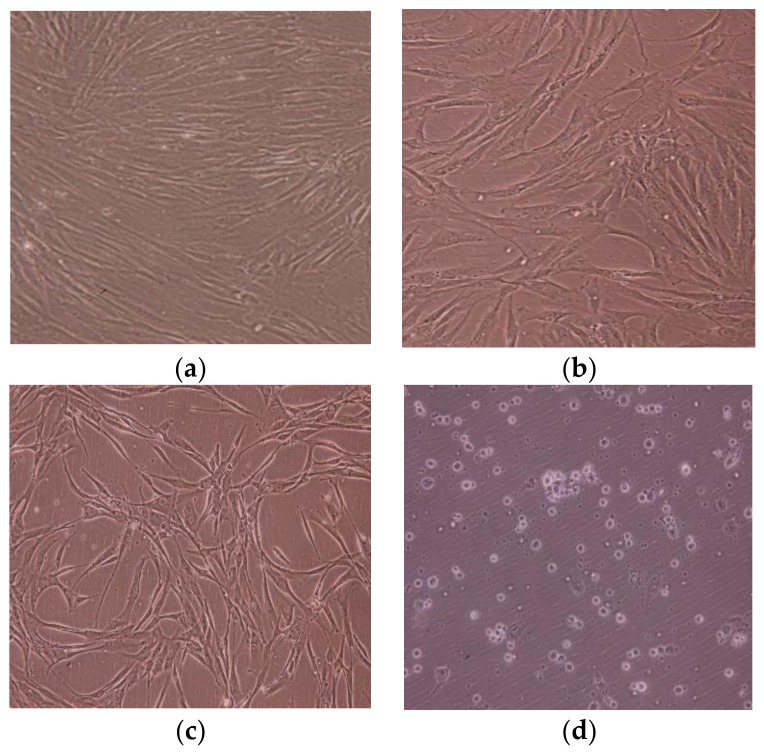
HSF cell growth in the (**a**) extraction medium and on the surface of the (**b**) Ti (uncoated) sample, (**c**) TiV coating, and (**d**) 700 °C HTA Ti-V-O coating.

**Figure 15:**
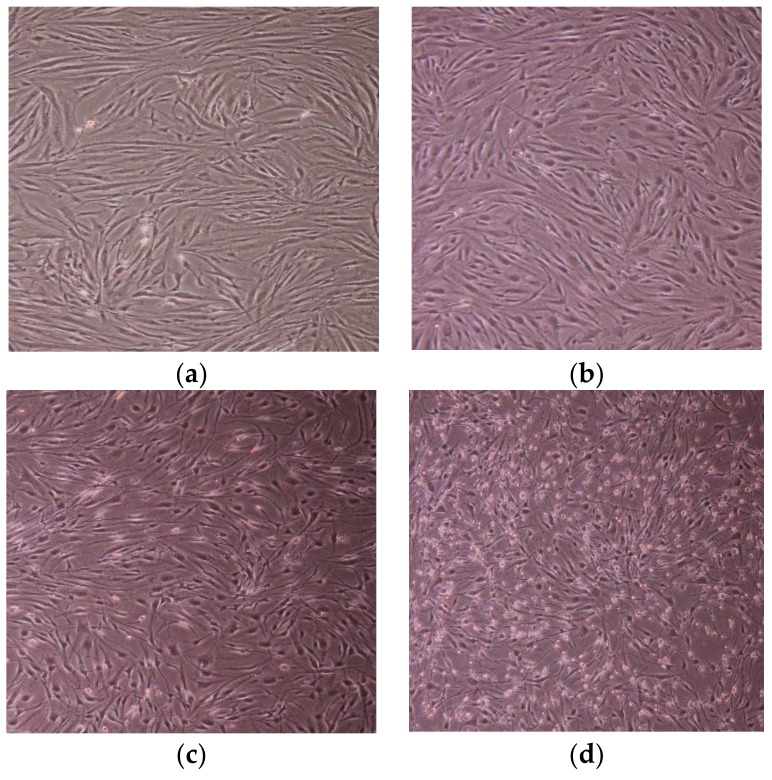
HSF cell growth in the (**a**) extraction medium and on the surface of the (**b**) Ti (uncoated) sample, (**c**) TiV coating, and (**d**) 100 kHz LSTO Ti-V-O coating.

Figure 14 and Figure 15 show cell shape and number during the cytotoxicity test, and show that both the HTA-deposited coating and LSTO Ti-V-O coating demonstrated poor cell proliferation. Not only was the cell quantity of the Ti-V-O coating poor, but the cells were also loosely attached to the sample. The present study verified that the Ti-V-O coating exhibited excellent antibacterial properties, and the vanadium compounds within the Ti-V-O coating induced inflammation in cells. Some researchers have indicated that oxidation states of vanadium could induce DNA damage [21,22] in cells, and excessive amounts of vanadium compounds exert observable toxic effects on body tissues [23]. Although vanadium has been confirmed as a toxic transition metal, the application of it, in cancer treatment and as a novel medicine in special compounds against viral infections, such as Dengue fever, SARS (severe acute respiratory syndrome), and HIV (human immunodeficiency virus) [24], still shows great potential for the future. 

## 4. Conclusions

This study used a cathodic arc deposition technique to deposit TiV thin films over pure titanium substrates. HTA and LSTO methods were then used to create Ti-V-O coatings on thin film samples. After the temperature of HTA was increased, the Ti-V-O coating exhibited needle-, stripe-, and grain-shaped surface textures. LSTO coating samples exhibited uniformly textured microstructures. Compared with other coatings created using the same method, the 700 °C HTA and 100 kHz LSTO Ti-V-O coatings had the most substantial surface hardness and lowest COF. The HTA Ti-V-O thin films exhibited higher wettability when annealed with higher temperatures. However, the surface of the 100 kHz LSTO Ti-V-O thin film sample demonstrated the lotus leaf-like effect, exhibiting superhydrophobic properties (140° water contact angle).

Both the HTA and LSTO Ti-V-O coatings exhibited noticeable antibacterial properties. Since the 100 kHz LSTO Ti-V-O coating also demonstrated superhydrophobic properties, it consisted of superior antibacterial properties compared with other LSTO coatings. The 700 °C HTA and 100 kHz LSTO Ti-V-O coatings demonstrated noticeable cytotoxicity and low cell proliferation. In summary, the HTA and LSTO Ti-V-O coatings introduced in this study are promising candidates for the surface modification of Ti, with the potential ability to combine the antibacterial properties with tribological performance in the biomedical field. Since the Ti-V-O coatings exhibited strong antibacterial effects and high surface hardness, future applications of Ti-V-O coatings should be targeted toward medical instruments that do not come into contact with body tissues. 

## Figures and Tables

**Table 1:** Energy dispersive spectroscopy (EDS) element mapping results of the TV coatings and the Ti-V-O coatings following HTA.

Element	TiV	Ti-V-O 500 °C	Ti-V-O 600 °C	Ti-V-O 700 °C
Ti (at. %)	41.30	25.34	10.41	31.29
V (at. %)	58.70	36.36	32.38	4.28
O (at. %)	-	38.30	57.21	64.43

**Table 2:** EDS element mapping results of the TV coatings and the Ti-V-O coatings following LSTO.

Element	TiV	Ti-V-O 100 kHz	Ti-V-O 300 kHz	Ti-V-O 500 kHz
Ti (at. %)	41.30	35.55	34.69	39.59
V (at. %)	58.70	12.27	15.64	23.40
O (at. %)	-	52.18	49.67	37.01
